# Ectomycorrhizal fungal community structure in a young orchard of grafted and ungrafted hybrid chestnut saplings

**DOI:** 10.1007/s00572-020-01015-0

**Published:** 2021-01-27

**Authors:** Serena Santolamazza-Carbone, Laura Iglesias-Bernabé, Esteban Sinde-Stompel, Pedro Pablo Gallego

**Affiliations:** 1 Applied Plant & Soil Biology, Plant Biology and Soil Science Department, Biology Faculty, University of Vigo, E-36310 Vigo, Spain; 2 CITACA - Agri-Food Research and Transfer Cluster, University of Vigo, Ourense, Spain; 3 Hifas Foresta SL, Portamuiños 7, Bora, 36154 Pontevedra, Spain

**Keywords:** *Castanea sativa* × *castanea crenata*, Early-stage fungi, Ectomycorrhizal colonization rate, Internal transcriber spacer (ITS) regions, Grafting, Fungal diversity

## Abstract

Ectomycorrhizal (ECM) fungal community of the European chestnut has been poorly investigated, and mostly by sporocarp sampling. We proposed the study of the ECM fungal community of 2-year-old chestnut hybrids *Castanea* × *coudercii* (*Castanea sativa* × *Castanea crenata*) using molecular approaches. By using the chestnut hybrid clones 111 and 125, we assessed the impact of grafting on ECM colonization rate, species diversity, and fungal community composition. The clone type did not have an impact on the studied variables; however, grafting significantly influenced ECM colonization rate in clone 111. Species diversity and richness did not vary between the experimental groups. Grafted and ungrafted plants of clone 111 had a different ECM fungal species composition. Sequence data from ITS regions of rDNA revealed the presence of 9 orders, 15 families, 19 genera, and 27 species of ECM fungi, most of them generalist, early-stage species. Thirteen new taxa were described in association with chestnuts*.* The basidiomycetes Agaricales (13 taxa) and Boletales (11 taxa) represented 36% and 31%, of the total sampled ECM fungal taxa, respectively. *Scleroderma citrinum*, *S. areolatum*, and *S. polyrhizum* (Boletales) were found in 86% of the trees and represented 39% of total ECM root tips. The ascomycete *Cenococcum geophilum* (Mytilinidiales) was found in 80% of the trees but accounted only for 6% of the colonized root tips. These results could help to unveil the impact of grafting on fungal symbionts, improving management of chestnut agro-ecosystems and production of edible fungal species.

## Introduction

The chestnuts are deciduous trees and shrubs belonging to the genus *Castanea* Mill. (Fagaceae), a small taxon which includes 13 species. Native from temperate countries of the Northern Hemisphere, the chestnut is widely distributed in southern Europe, eastern North America, northern Africa, Asia Minor, and eastern Asia (Conedera et al. 2016). Four species are mainly used for commercial production (Hardin et al. 2001): *C. sativa* Mill. (European chestnut), *C. crenata* Sieb. and Zucc. (Japanese chestnut), *C. mollissima* Bl. (Chinese chestnut), and *C. dentata* (Marsh.) Borkh. (American chestnut).

Chestnut cultivation was initially developed by the ancient Greeks, and successively propagated in the rest of Europe by the Romans as a crop, by grafting (Conedera et al. 2004). Thus, *C. sativa*, the only native species of the European continent, covering more than 2.5 million hectares, is mainly distributed among France, Italy, Spain, Greece, Portugal, and Switzerland (Conedera et al. 2016; Roces-Díaz et al. 2018).

Chestnut forest stands and orchards are part of the traditional and historical landscape and are amongst the conservation priorities in Europe (European Council 1992). Chestnut orchards have a notable multipurpose character, producing not only timber, firewood, forage, and tasty edible fruits but also secondary products such as pasture, hay, mushrooms, and honey (Pereira-Lorenzo et al. 2010). In Spain, chestnut orchards occupy 272,400 ha, being the dominant species in 154,500 ha (MAPAMA 2013). Galicia (NW Spain) is the main producing area in the country, followed by Castilla-León (El Bierzo), Central Spain (Salamanca, Cáceres, and Ávila), Andalucía, and Canary Islands (Tenerife, La Palma, Gomera, and El Hierro).

In Galicia, both forest stands and cultivated orchards cover a total surface area of 45,518 ha (Xunta de Galicia 2001). Although chestnut groves are rarely intercropped or grazed, such groves create a mosaic of land uses including orchard, coppice, and forests. Great part of the adult chestnut areas belongs to the Natura 2000 network, being an important site for birds, and also included in the recovery plan for bear populations in Galicia. Also, the chestnuts produced in this region are recognized as protected geographical indication (PGI) and are exported to selective markets in Europe (Fernández-López 2013).

At the end of the 19th century, native *C. sativa* was dramatically threatened by the spread of ink disease, caused by *Phytophthora cambivora* and *P. cinnamomi*, and more recently by the chestnut blight, caused by *Cryphonectria parasitica* (Bouhier 1979; Mansilla Vázquez et al. 2000; Miranda-Fontaiña et al. 2007). *Castanea sativa* was traditionally the main species in Spain´s chestnut orchards, but in Galicia since the 1950s, interspecific hybrids, such as *Castanea* × *coudercii* (*Castanea sativa* × *Castanea crenata*), have been used as rootstock for grafting *C. sativa* plants (Fernández-López 2011). In addition, hybrids of *C. sativa* with *C. crenata* and *C. mollisima* offered tolerance and/or resistance to ink diseases, allowing to overcome the infection (Miranda-Fontaiña et al. 2007; Fernández-López 2011). The selection of chestnut hybrids which are resistant to blight or ink diseases, and the necessity of propagating them asexually, led to an important commercial production of selected hybrid clones.

The vegetative propagation used for woody plants are grafting (sometimes by budding), cuttings from stems and roots, layering, and micro-propagation (Macdonald 1990). However, grafting has proven to be a more successful and feasible method for asexual propagation (Huang et al. 1994). Today, chestnut orchards for fruit production consist of open stands, generally composed of grafted trees because of the self-sterility of some chestnut cultivars. It is accomplished most commonly by connecting two plant segments, the shoot piece called “scion” and the root piece called “rootstock,” generally selected for disease resistance. It has been discovered from experiments that grafting influences absorption and translocation of phosphorus, nitrogen, magnesium, and calcium (Ikeda et al. 1986; Ruiz-Medrano et al. 1999; Pulgar et al. 2000), improves nutrient uptake and increases photosynthesis (Hu et al. 2006). Repeated grafting improved the in vitro culture capacity of a recalcitrant mature chestnut tree, indicating that reinvigoration increases rooting capacity (Giovannelli and Giannini 2000; Fernández-Lorenzo and Fernández-López 2005; Fernández-Lorenzo and Crecente 2010). Here, we hypothesize that grafted chestnuts may experience a better performance, which could be translated into an increase of ECM fungal species richness and colonization rate, through a higher allocation of carbohydrates to the fungal symbionts.

Naturally, *C. sativa* trees establish ectomycorrhizal (ECM) associations with a large macro-fungal community, usually dominated by the families Amanitaceae, Boletaceae, and Cortinariaceae, being the genera *Amanita*, *Cortinarius*, *Inocybe*, *Lactarius*, *Russula*, and *Tricholoma*, the most abundant taxa above-ground, in terms of species number (Baptista et al. 2010; Martins et al. 2011; Álvarez-La Fuente 2015; Baptista et al. 2015)*.* Positive effect of the host plant-fungus symbiosis, such as an increased phosphorus and nitrogen uptake, increased growth (Martins et al. 1996; Martins et al. 1997) and better protection against root pathogens have been assessed (Branzanti et al. 1999). However, during a long time, chestnut orchard planning and management has paid little attention to the ECM fungal diversity and to the harvesting of wild edible fungi. Recently, because of a decline in forest-based industries in some countries, the collection and commercialization of marketable edible mushrooms represent an important income associated with chestnut orchards (Román and Boa 2006). In particular, macro-fungal species such as *Amanita cesarea (Scop.) Pers.*, *Cantharellus cibarius* Fr., *Boletus fragans*, and *Boletus edulis* complex (*B. edulis* Bull.: Fr. *sensu stricto*; *B. aereus* Bull.: Fr.; *B. pinophilus* Pilat et Dermek; and *B. reticulatus* Schaeff), are especially appreciated (Leonardi et al. 2005). Despite the ecological and economic importance of the fungal assemblage associated with *C. sativa*, literature on this topic is still scarce.

The ECM fungal community associated with chestnut orchards has been investigated, mostly by fruit bodies identification, in some European countries such as Italy (Laganà et al. 2002; Peintner et al. 2007; Blom et al. 2009; Ambrosio and Zotti 2015), Greece (Diamandis and Perlerou 2001; Polemis et al. 2011), Portugal (Baptista et al. 2010; Martins et al. 2011; Baptista et al. 2015; Reis et al. 2016), Romania (Chira and Chira 2003), and Spain (Álvarez-La Fuente 2015). Although above-ground surveys of epigeous fungal species provide valuable information on the reproductive investment of co-occurring species, they only partially reflect fungal diversity and represent a distorted view of the distribution of soil mycelia (Gardes and Bruns 1996; Straatsma et al. 2001). Studies performed in chestnut orchards have revealed little overlap between above- and below-ground fungal communities by using fruit body surveys and internal transcriber spacer (ITS) barcoding of ECM root tips (Peintner et al. 2007; Baptista et al. 2015).

Here, we proposed for the first time (1) the study of the ECM fungal community of young chestnut hybrid *Castanea* × *coudercii* saplings in NW Spain, by using a below-ground molecular approach, and (2) the evaluation of the impact of chestnut hybrid clone type and grafting on ECM colonization rate, fungal community composition, importance value (sum of relative frequency and relative abundance), richness, and diversity.

## Material and methods

### Study area and orchard characteristics

The study was conducted in Galicia, which is the most important forestry region of Spain (MAPAMA 2013). It is characterized by an Atlantic humid climate without long frost periods and with a uniformly distributed annual precipitation (Martínez Cortizas and Pérez Alberti 2000). The average annual temperature varies between 8 and 14 °C. Annual rainfall ranges from 600 to 2500 mm, with some sites over 3000 mm per year. The experimental orchard is located at Bora, Pontevedra province (42° 25′ 56.5″ N–8° 34′ 41″ W) and belongs to Hifas Foresta company. This is a 3-year-old, productive orchard, irrigated 4/5 times per year and fertilized with Ficosagro, a liquid bio-stimulant. This product is rich in microorganisms (Agarophytae algae, sulfate-reducing bacteria, aerobic mesophilic bacteria, lactic acid bacteria, yeast, and molds) and improves the recovery of soil microflora and microfauna, and helps to release soil nutrients such as nitrogen and phosphorus that can be absorbed by the roots of the plants. The experimental site is surrounded by pastures, fruit orchards, mature chestnuts, and oak trees (*Quercus robur*).

### Soil characterization

In Galicia, soils are typically acidic and sandy; they contain a high proportion of coarse fragments and have a high content of organic matter and low levels of nutrients. Granite, schist, and slate are the dominant parent materials, leading to ranker-like soils, litosols, or humid cambisols (Macías and Calvo de Anta 2008). The studied area is characterized by soils with a predominance of the sand fraction and little amount of clay, high porosity, and low water retention capacity. Soil sampling was carried out in October 2018, to assess the main chemical characters of the studied orchard. In the orchard, twelve soil samples 5 × 5 cm were taken in the first horizon (0–5 cm deep) with a soil corer, and another twelve soil samples at 20 cm deep. Soil analysis (pH, organic matter (%), organic C, available soil N, cation exchange capacity (CEC), and Ca, K, Mg, Na, Al) was carried out at the Plant Biology and Soil Sciences Department of the University of Vigo and is summarized in the Electronic Supplementary Material S1.

### Experimental design and root sample collection

Young (2-year-old) chestnut clones, named 111 and 125, of the hybrid *Castanea* × *coudercii* from Hifas Foresta germplasm, were used as plant material. One half of the chestnut clone 111 was grafted with Bouche de Bétizac cultivar, which is a French controlled hybrid between *C. sativa* and *C. crenata*, mostly appreciated for its large fruits. Similarly, one half of the chestnut clone 125 was also grafted with the *C. sativa* cultivar “Judía” which is the main cultivar in north-western Trás-os-Montes, and quite popular in Portugal for its fruit yield (Dinis et al. 2009). The other halves of both chestnut clones were kept ungrafted.

In the orchard, mother plants were induced to produce clones by layering. The clones were cultivated during the winter and successively grafted in spring 2017. The rootstocks obtained were grafted following the English double protocol (i.e., whip and tongue graft of scion and rootstock, with large contact between both cambial surfaces). Grafted and ungrafted plants were 30 cm apart along the same row. The rows were 50 cm apart. A total of 16 saplings of chestnut clone 111 grafted (*n* = 8) and ungrafted (*n* = 8) and another 16 saplings of the chestnut clone 125 grafted (*n* = 8) and ungrafted (*n* = 8) were sampled for fungal root tip analysis. Root samples were collected during January–February 2018. The below-ground fungal community of the experimental orchard was studied following the procedure of Pestaña-Nieto and Santolamazza-Carbone (2009). Approximately 50 cm of fine roots (< 2 mm diameter) per tree were collected. Fine roots were cut with scissors, introduced in labelled plastic bags and transported to the laboratory, where they were stored at 4 °C until processing. Following Agerer (1987–2002), all ECM root tips were sorted into broadly defined morphotypes based on morphological characteristics, including color, shape, texture, ramification type, occurrence of cystidia, mantle type, emanating hyphae and rhizomorphs. Care was taken to separate morphotypes when slight variations were found. Finally, for each tree, subsamples of the morphotypes (depending on its abundance, it ranged from 10 to 100 mg) were placed individually in a 1.5 ml Eppendorf tube and stored at − 20 °C for molecular analysis, in order to provide taxonomic identification.

### Ecological parameters

Fifty pieces (approximately 1 cm) of roots were examined per tree. The fragments were randomly dispersed in a 9-cm-diameter Petri dish with grid lines in order to calculate the ECM colonization rate (proportion of ECM root tips/total root tips of 50 cm of root length) by using the gridline intersect method under a dissecting microscope (Brundrett et al. 1996). Once obtained the taxonomic identity of the ECM root tips, after molecular verification, ECM fungal community composition was assessed through the estimation of the relative abundance per plant (root tips produced by each taxon/ECM root tips of 50 cm of root length), the absolute frequency (no. of plants with a taxon/total sampled plants), and the relative frequency (absolute frequency of a taxon/Ʃ absolute frequency of all taxa) of each fungal species per host plant, were calculated. In addition, following Brundrett et al. (1996), we used the sum of relative frequency and relative abundance to obtain the importance value of each taxon in the ECM fungal community. Species richness was defined as the number of ECM species recorded per plant. Shannon–Wiener diversity index (H’) was used to characterize the composition of the ECM fungal communities across the experimental groups, by using the software EstimateS 9.1.0 (Colwell 2006). The Shannon–Wiener index increases when the number of taxa increases or the distribution of the species becomes more even.

### Molecular analysis

The taxonomic identity of the morphotypes was ascertained by PCR amplification and direct sequencing of the ITS regions (Horton and Bruns 2001). The samples were amplified by using the direct PCR procedure (Iotti and Zambonelli 2006). This procedure, which is faster and less expensive than conventional methods, allows to amplify ITS fragments directly from the root tips, without performing the previous DNA extraction and purification. ECM root tips manipulation was carried out in Petri dishes containing sterile distilled water under a dissecting microscope (× 20). A small portion of the ECM mantle was peeled with a sterilized surgical blade and directly transferred to the PCR tube containing pure sterilized water and BSA. For this method, the PCR reaction protocol was as follows: 95 °C for 6 min, followed by 30 cycles of 94 °C for 30 s, 55 °C for 30 s, and 72 °C for 10 min. However, direct PCR may not always provide a good amplification of dark pigmented ECM root tips or with recalcitrant DNA. In these cases, fungal DNA was extracted from fresh ECM root tips using the EZNA Fungal DNA Kit (Omega Bio-Tek, USA). The eluted DNA was successively purified with the Power Clean Pro DNA Clean up Kit (MoBio Laboratories Inc.) according to the manufacturer’s instructions. PCRs were conducted in 30 µl volume reaction containing MgCl_2_ 50 mM (BIORAD), DreamTaq Green PCR Master Mix (2×) (Thermo Scientific), bovine serum albumin (BSA) 10× (Thermo Scientific), pure water nuclease-free (Thermo Scientific), and 2 µl of undiluted DNA (concentration ranged between 50 and 100 ng/µl). The universal primers ITS1/ITS4 and ITS1F/ ITS4B were used to amplify the ITS-1, 5.8S, and ITS2 regions of the nuclear rDNA. The amplification conditions were optimized for each primer combination, but the general reaction protocol was as follows: 94 °C for 3 min, followed by 40 cycles of 94 °C for 30 s, 55 °C for 30 s, 72 °C for 1 min and a final extension step of 72 °C for 10 min. A sample of 3 µl of each PCR reaction was electrophoresed in a 2% agarose gel and visualized with the UV-transilluminator. Negative controls without DNA were performed, in order to detect possible contaminations.

PCR products were purified and sequenced by Macrogen Laboratories (http://www.macrogen.com). Forward and reverse sequences were aligned and edited using BioEdit Sequence Alignment Editor 7.2.6 (http://www.mbio.ncsu.edu/bioedit/bioedit.html). All sequences were identified to genus or species level by querying the GenBank database, using the nucleotide-nucleotide (blastn) BLAST search option, available through the National Center for Biotechnology Information (NCBI). We considered only sequences with 80–100% of query coverage and E value (expectation value) equal to zero. Assignment to taxonomic categories was performed by using the following criterion: sequences with similarity between 90 and 97% were identified to genus groups, while sequence similarity ≥ 98% gives a match for species identification.

The nomenclature of fungal taxa and the geographical distribution of these species have been assessed by using the Index Fungorum (www.indexfungorum.org) and the global mapper provided by the online encyclopedia of life www.discoverlife.org and the Global Biodiversity Information Facility web (www.gbif.org), respectively.

### Statistical analysis

Prior the analyses, variables using continuous data were checked for normality by Kolmogorov–Smirnov test and Bartlett’s test for homogeneity of variances. Variables using binomial data (such as ratios expressed in percentage) were previously subjected to logarithm (*x* + 1) transformation.

The impact of chestnut clone and grafting on ECM colonization rate and fungal species richness were investigated by two-way ANOVA. Pairwise comparisons were done with LSD (least significant difference) Fisher’s test at level of significance α = 0.05. The analyses were performed with GenStat release 10.2 (VSN International, Hemel Hempstead, UK).

To estimate the species accumulation curve index (S), we used Chao2 estimators. Species accumulation curves with 95% confidence intervals and 100 permutations were computed to assess the efficiency of ECM sampling for each experimental group and were performed with EstimateS 9.1.0 (Colwell 2006).

To test for differences in ECM fungal species composition and to estimate the components of variation, we used the permutational multivariate analysis of variance (PERMANOVA), with the number of root tips of each ECM taxon as the variables. The plant clone identity and the grafting were used as fixed factors. PERMANOVA analyses were based on Bray–Curtis similarities. We used PRIMER 7.0.13 with the PERMANOVA + 1 add for these analyses. The analysis was performed using 9999 permutations, with permutation of residuals under a reduced model, fixed effects sum set to zero and type III sums of squares. Significance was declared at α = 0.05.

## Results

A total of 25,296 chestnut hybrid root tips were examined under the dissecting microscope, of which 3,784 were ECM root tips, initially sorted among 382 morphotypes. The 48% of the samples were successfully amplified with the direct PCR technique.

**Fig. 1:**
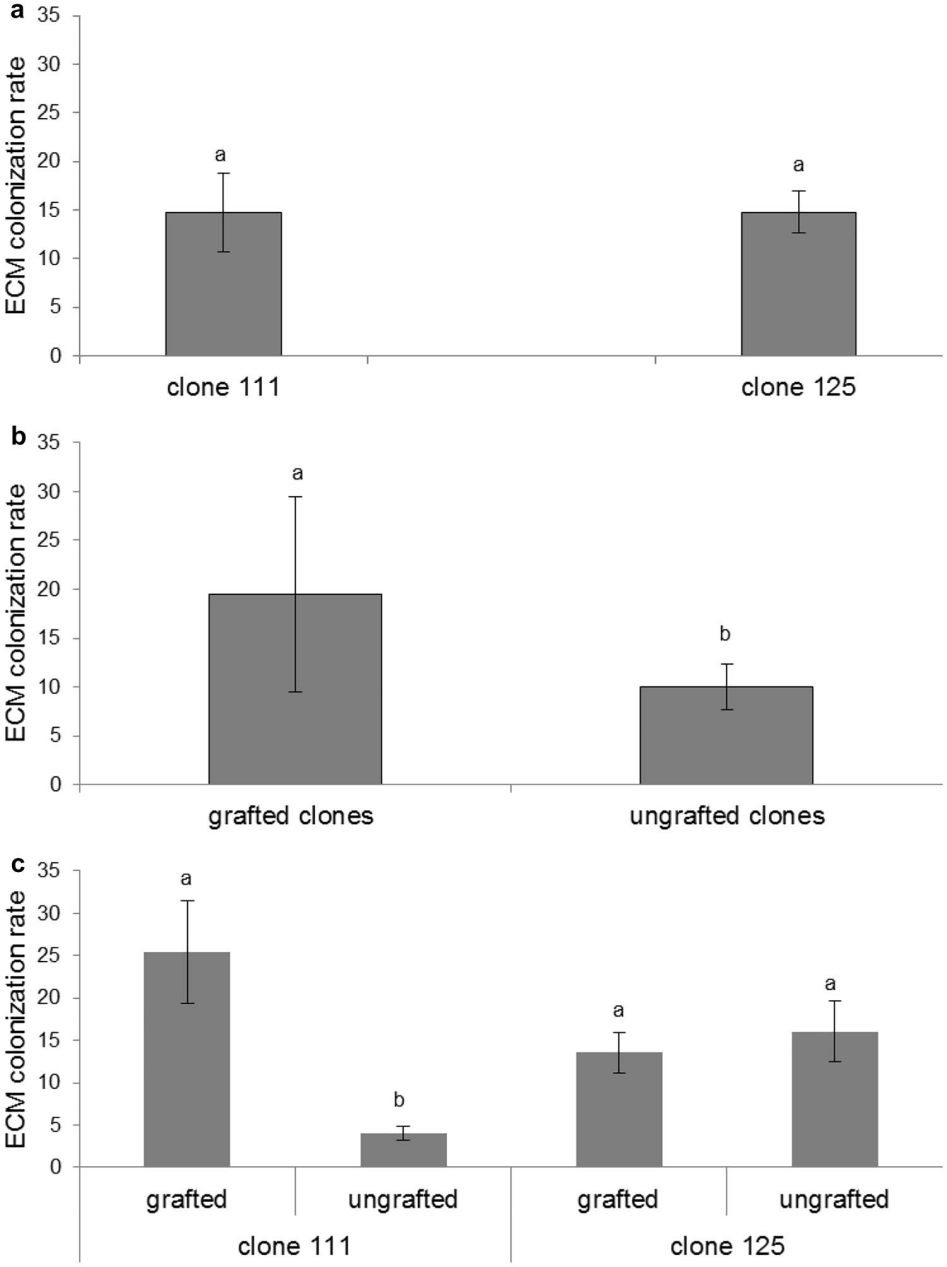
ECM colonization rate (proportion of ECM root tips/total root tips of 50 cm of root length) calculated for chestnut hybrid clones 111 and 125 irrespective of grafting **a** for grafted and ungrafted chestnut hybrids irrespective of clone type **b** and for chestnut hybrid clones 111 and 125 grafted and ungrafted **c** Different letter means the existence of significant differences at α = 0.05

The chestnut hybrid clone type (111 vs. 125) did not have any impact on ECM colonization rate (*F*_1,31_ = 2.03, *P* = 0.165) (Fig. 1a). When data from both clones were pooled, ECM colonization was greater in grafted than in ungrafted saplings (*F*_1,31_ = 12.75,* P* = 0.001) (Fig. 1b). With chestnut clone 111, grafted plants had a higher ECM colonization rate than the ungrafted ones (*F*_1,31_ = 29.20, *P* < 0.001), whereas such difference was not found in the chestnut clone 125 (*F*_1,31_ = 0.04, *P* = 0.845) (Fig. 1c). On the other hand, among ungrafted plants, the chestnut clone 125 had a higher ECM colonization rate than clone 111 (*F*_1,15_ = 12.20, *P* = 0.004) (Fig. 1c). ECM colonization rate, however, did not vary between the grafted clones 111 and 125 (*F*_1,15_ = 3.46, *P* = 0.084). Neither clone type (*F*_1,31_ = 0.21, *P* = 0.654) nor grafting (*F*_1,31_ = 0.57, *P* = 0.457), influenced ECM species richness. Similarly, species richness did not vary within clone 111 (*F*_1,31_ = 2.34, *P* = 0.150) and clone 125 (*F*_1,31_ = 0.25, *P* = 0.626) when comparing grafted and ungrafted plants, and also between the grafted clones 111 and 125 (*F*_1,15_ = 0.07, *P* = 0.792).

Anatomical features and sequence data from ITS regions of rDNA revealed the presence of 9 orders, 15 families, 19 genera, and 27 species of ECM fungi (Table 1). Among the identified ECM fungal taxa, 13 were not previously reported in association with chestnuts (Table 1). Moreover, *Hebeloma arenosum*, *Hymenogaster huthii*, *Laccaria montana*, and *Xerocomellus ripariellus* have been identified for the first time in Spain. In addition, *Hortiboletus rubellus*, *Inocybe curvipes*, *I. soluta*, *Laccaria macrocystidiata*, *L. proxima*, *Sphaerosporella brunnea*, *Xerocomus pruinatus*, and *X. rubellus* have been detected for the first time in Galicia.

**Table 1:** ECM fungal species found in association with clones 111 and 125 of the chestnut hybrid *Castanea* × *coudercii*. Taxonomical identity was ascertained by direct sequencing of the ITS regions and BLAST search. Species in bold are found for the first time in association with chestnut. *Cenococcum geophilum* was not subjected to molecular analysis because its ECM root tips can be easily identified by morphological features (i.e., black mantle with star-patterned synenchyma and stiff, black, emanating hyphae).

Order	Family	Identification (Blast search)	Similarity (%)	Size (bp)	GenBank accession
Agaricales	Cortinariaceae	*Cortinarius* sp. (FJ157140)	99	764	MN651968
	Hymenogastraceae	***Hebeloma arenosum*** (KX215469) *	99	788	MN651989
	Hymenogastraceae	*Hebeloma* sp. (EF564171)	99	714	MN651990
	Hymenogastraceae	***Hebeloma sacchariolens*** (JX030286)	99	820	MN651991
	Hymenogastraceae	***Hymenogaster huthii*** (GU479240) *	99	840	MN652640
	Inocybaceae	***Inocybe curvipes*** (AM882813) **	99	779	MN652657
	Inocybaceae	*Inocybe maculata* (AM882959)	99	793	MN654126
	Inocybaceae	***Inocybe soluta*** (HQ604553) **	99	768	MN663128
	Inocybaceae	*Inocybe* sp. (KM576446)	99	791	MN663131
	Hydnangiaceae	***Laccaria macrocystidiata ***(KM067850)**	99	568	MN663129
	Hydnangiaceae	***Laccaria montana*** (EU486434) *	99	794	MN663132
	Hydnangiaceae	***Laccaria proxima*** (JX907813) **	99	778	MN663149
	Hydnangiaceae	*Laccaria* sp. (AJ534899)	99	789	MN663158
Boletales	Boletaceae	*Boletus aereus* (MH011927)	98	858	MN652653
	Boletaceae	*Boletus reticulatus* (KC261837)	99	698	MN649213
	Boletaceae	***Hortiboletus rubellus*** (KX438318) **	99	586	MN652008
	Boletaceae	***Xerocomellus ripariellus*** (KV355482) *	99	853	MN685108
	Boletaceae	*Xerocomellus cisalpinus* (HM190077)	99	597	MN685109
	Boletaceae	***Xerocomus pruinatus*** (AF402140) **	99	868	MN696797
	Boletaceae	*Xerocomus rubellus* (JQ685725) **	99	758	MN685116
	Diplocystaceae	*Astraeus hygrometricus* (KY96000)	99	838	MN649186
	Sclerodermataceae	*Scleroderma areolatum* (FM213352)	99	817	MN684210
	Sclerodermataceae	*Scleroderma citrinum* (KJ679576)	99	709	MN663268
	Sclerodermataceae	*Scleroderma polyrhizum* (KY693661)	98	716	MN663271
Cantharellales	Clavulinaceae	*Clavulina cristata* (KP454011)	98	789	MN649216
	Clavulinaceae	*Clavulina* sp. (MH040298)	99	705	MN651571
	Hydnaceae	*Sistotrema* sp. (KF218968)	90	762	MN683569
Mytilinidiales	Gloniaceae	*Cenococcum geophilum*			
Pezizales	Pyronemataceae	*Sphaerosporella brunnea* (KC008078) **	99	508	MN683734
Russulales	Russulaceae	*Russula amoenolens* (MG679814)	99	766	MN663161
	Russulaceae	*Russula parazurea* (DQ422007)	98	759	MN663162
Sebacinales	Sebacinaceae	*Sebacina* sp. (JQ711843)	97	779	MN684321
Thelephorales	Thelephoraceae	***Thelephora terrestris*** (JQ711981)	99	748	MN683732
	Thelephoraceae	***Tomentella sublilacina*** (KP783476)	98	729	MN683827
	Thelephoraceae	*Tomentella* sp. (KY694394)	99	762	MN683825
Trechisporales	Hydnodontaceae	*Trechispora* sp. (KX058343)	100	799	MN684322

*ECM fungal species identified for the first time in Galicia and new for Spain

**ECM fungal species identified for the first time in Galicia

All the ECM fungal taxa were basidiomycetes (Agaricales, Boletales, Cantharellales, Russulales, Sebacinales, Thelephorales, and Trechisporales), with the exception of *Cenococcum geophilum* (Mytilinidiales) and *Sphaerosporella brunnea* (Pezizales), both ascomycetes (Table 1). The order groups with the highest species richness were Agaricales (13 taxa) and Boletales (11 taxa), which represented 36% and 31%, of total sampled ECM fungal taxa, respectively. Root tips colonized by Agaricales represented only 10% of the 3784 sampled ECM root tips, whereas Boletales were detected in 48% of the ECM root tips. *C. geophilum* was found in 80% of the experimental trees but Mytilinidiales accounted only for 6% of colonized root tips.

**Fig. 2:**
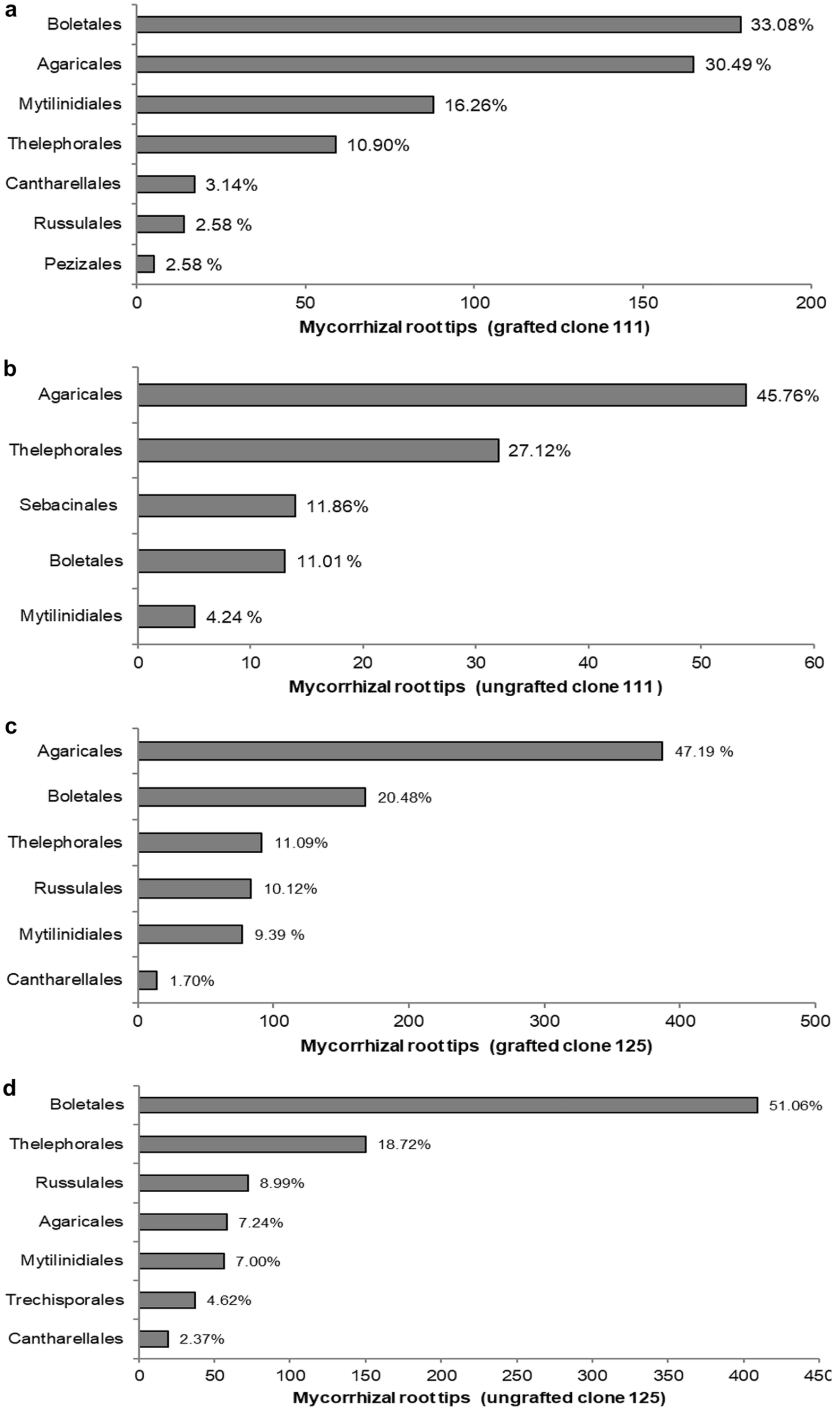
Ranked ECM fungal orders according to their number of mycorrhizal root tips for chestnut clone 111 grafted **a** and ungrafted **b** and for chestnut clone 125 grafted **c** and ungrafted **d**. Percentages at the end of the bars show the relative abundance, calculated as number of root tips for each fungal order/number of root tips per experimental group × 100

*Scleroderma* spp. (*S. areolatum*, *S. citrinum*, and *S. polyrhizum*) were found in 86% of the sampled trees and represented 39% of sampled ECM root tips. Percentages of relative abundance calculated for each fungal order inside each experimental group are shown in Fig. 2a–d. For the grafted chestnut clone 111, Boletales (33.08%) and Agaricales (30.49%) dominate the fungal community, whereas Agaricales (45.76%) and Thelephorales (27.12%) are the most abundant orders for the ungrafted clone 111 (Fig 2a, b). A similar trend was found for the grafted chestnut clone 125, where Agaricales (47.19%) and Boletales (20.48%) are the most abundant taxa inside the fungal community (Fig. 2c). For the ungrafted chestnut clone 125, Boletales had the highest abundance (51.06%), followed by Thelephorales (18.72%) (Fig. 2d)

**Fig. 3:**
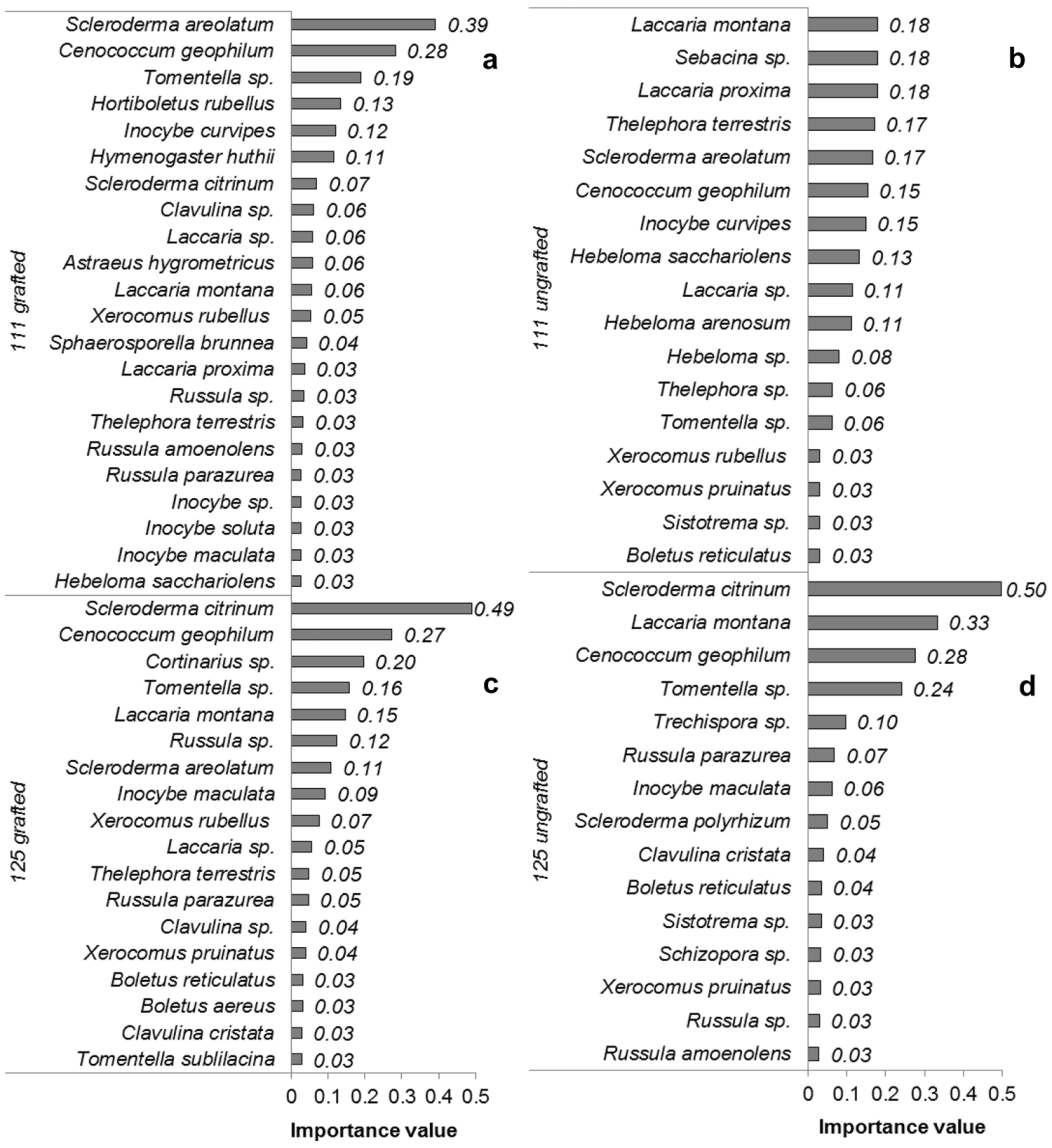
ECM fungal taxa associated with the grafted and ungrafted chestnut clone 111 **a**, **b** and the grafted and ungrafted chestnut clone 125 **c**, **d**. The bars represent the sum of relative frequency and relative abundance, which provides the importance value of each taxon inside the fungal community

The ranking of importance values of the identified ECM fungal taxa is shown in Fig. 3a–d. *Scleroderma* spp. appeared in the first position of the importance value ranking of three out of the four experimental treatments, followed by *C. geophilum* and *Laccaria* spp*.* The ECM fungal community associated with the ungrafted clone 111 (Fig. 3b), was characterized by a group of 6–7 taxa with similar importance value, being impossible to indicate a really dominant taxon, as it occurred in the other experimental groups.

Although, ECM fungal species (73%) dominated the below-ground fungal community (19 genera, and 27 species), also non-ECM fungi (ericoid, saprotrophic, and wood decay fungi) were detected, representing 27% (12 genera and 9 species) of identified taxa (Electronic Supplementary Material S3). Non-ECM fungi, however, were excluded from further analyses.

Rarefaction curves for ECM root tip surveys did not reach a plateau (Electronic Supplementary Material S2). The ratio between the observed and the estimated ECM species richness (using the Chao2 estimators) ranged between 25 and 80%, with the lower ratios corresponding to ungrafted clone 125, which suggest some under-sampling (Electronic Supplementary Material S2).

Shannon–Wiener diversity index (H’) did not vary among chestnut clone types (*F*_1,31_ = 0.00, *P* = 0.945), and between grafted and ungrafted plants (*F*_1,31_ = 0.54, *P* = 0.469). The similarity of ECM fungal composition was significantly affected by the host plant clone identity but not by grafting (PERMANOVA analysis; Table 2). There were differences in fungal composition between clone 111 and 125 (up to eleven ECM fungal species were found exclusively in clone 111 and 5 in clone 125) and within clone 111 between grafted and ungrafted plants, although this effect was not detected within clone 125 (Table 2). In addition, 6 ECM fungal species (*Hortiboletus rubellus*, *Hymenogaster huthii*, *Astraeus hygrometricus*, *Sphaerosporella brunnea*, *Laccaria proxima*, and *Inocybe solute*) were found exclusively in the grafted chestnut clone 111, whereas only 2–3 exclusive ECM fungal species were recorded in the other treatment groups (Fig. 3a–d).

**Table 2:** Results of the PERMANOVA analysis to test for significant changes in ECM fungal taxa composition between plant clones 111 and 125 and between grafted and ungrafted plants. The same test was repeated within each clone, between grafted and ungrafted plants

General	*df*	SS	MS	Pseudo-*F*	*P* (perm)
Clone	1	10714	10714	2.8233	0.001
Grafting	1	5502.5	5502.5	1.45	0.066
Residual	29	94868	3794.7		
Total	32	1.1513E+05			
Clone 111					
Grafting	1	6673.5	6673.5	1.6597	0.030
Residual	15	52273			
Total	16	58946			
Clone 125					
Grafting	1	1954	1954	0.86454	0.592
Residual	15	27122	2260.1		
Total	16	290076			

## Discussion

Grafting is an ancient procedure commonly used to improve growth, yield, and resistance to diseases of woody trees such as chestnut (Giovannelli and Giannini 2000; Fernández-Lorenzo and Fernández-López 2005; Fernández-Lorenzo and Crecente 2010). Many attempts to provide a satisfactory explanation of short- and long-term consequences of grafting have been made, but, at present, many physical, physiological, biochemical, and molecular mechanisms of this practice are still unclear (Goldschmidt 2014; Belmonte-Ureña et al. 2019). As far as we know, this is the first time that the relationship between grafting and ECM fungal community structure is investigated. We hypothesized that grafted plants should experience a higher ECM colonization rate, but actually this effect was found only in the chestnut clone 111 grafted with the Bouche de Bétizac cultivar, mostly determined by *Scleroderma areolatum* (Boletales), *Inocybe curvipes* (Agaricales), *Tomentella* sp. (Thelephorales), and *Cenococcum geophilum* (Mytilinidiales). Similarly, importance value indicates a dominance of *S. areolatum*,* C. geophilum*, and *Tomentella* sp. for this experimental group. Thus, the chestnut clone 111 seems to be more responsive to grafting in comparison with clone 125, because the higher association with ECM fungi, and because ungrafted plants of clone 125 showed the lowest colonization rate. Clone 111 thus appears as a promising type which may ensure, under favorable soil conditions, a better performance and development of the plant. On the other hand, grafted plants of clone 125 did not increase their alliance with ECM fungi, showing a similar ECM colonization rate than ungrafted plants or grafted plants of clone 111. Possibly, the increase of the colonization rate by the fungal partners, which in turn allows a higher water and nutrient uptake, may depend on a better performance of the plant, promoted by grafting*.* Of course, at present, this is just speculative and further studies are needed to unveil the role of grafting on fungal symbionts dynamics and plant development. About ECM fungal richness and diversity, however, we did not find significant differences between groups, possibly due to the young age of the chestnut clones, being our first hypothesis not supported. Despite the fungal communities of the experimental groups were dominated by generalist species, and despite richness and diversity did not vary, significant differences in ECM fungal species composition were found between clones 111 and 125, and within the clone 111. Then, the grafted clone 111 had not only a higher fungal colonization rate, in comparison with ungrafted plants, but also harbored a significantly different ECM fungal community. In particular, there is a shift of the dominant fungal order within clone 111: Boletales members are preponderant among grafted plants (33%), whereas Agaricales dominate the ECM fungal community of ungrafted ones (46%). Fungal species with thick mantles, extensive extra-matrical mycelia or rhizomorphs, and large and abundant sporocarps (high-biomass species) have a larger carbon requirement and may impose a greater cost on the plant host (Saikkonen et al. 1999). Under the hypothesis of an increased photosynthetic performance of the scion leaves in response to grafting (Fullana-Pericás et al. 2019), the presence of high-biomass fungal species, such as the Boletales *Scleroderma*, *Hortiboletus* and *Xerocomus*, could be explained. On the other hand, among the ungrafted plants, which may be not able to support expensive fungal symbionts due to lower carbon allocation, low-biomass fungi of the Agaricales order, such as *Laccaria*, *Inocybe*, and *Hebeloma* are more abundant.

To our knowledge, this is the first report on the mycobiota associated with 2-year-old *Castanea* × *coudercii* hybrids using molecular approaches. Among the identified fungal taxa, there are 13 species that have not been previously documented in symbiosis with chestnuts*.* This finding supports the evidence that molecular identification of ECM fungal root tips provides better information about fungal diversity than above-ground sporocarp surveys only (Gardes and Bruns 1996). Species producing hypogeous sporocarps such as *Hymenogaster huthii* would have never been detected by above-ground sporocarp sampling. The genus *Hymenogaster* is the most species-rich genus of false truffles and includes a number of rarely recorded taxa, for which molecular study are necessary (Stielow et al. 2011). Also, the ascomycete *C. geophilum*, a ubiquitous, generalist ECM fungus, can only be detected on root tips or as sclerotia into the soil (Trappe 1964; Douhan and Rizzo 2004). Other species such as *Thelephora* sp. and *Tomentella* sp., well represented in all the experimental chestnut clones, are easily missed in above-ground sporocarp surveys, due to their resupinate fruitbodies (Gardes and Bruns 1996). Both genera are often found in plant nursery soil and are considered as important ECM mycobionts of seedlings, especially after planting on former agricultural land (Hilszczańska and Sierota 2006).

A *Sebacina* sp. was found in symbiosis with the ungrafted chestnut clone 111 only, being in the second position in the ranked importance value for the ECM fungal community (Fig. 3b). Fungi in the Sebacinales order display transitions from saprotrophy to endophytism and to mycorrhizal life styles. *Sebacina* is another example of a previously overlooked and now ubiquitous root symbiont (Kühdorf et al. 2014). The same is true for the species of the ascomycetous genus *Meliniomyces*, which was listed here among the non ECM species (Table S1). *Meliniomyces *sp. have been previously detected with molecular tools in roots of conifers such as *Pinus pinaster* (Pestaña Nieto and Santolamazza-Carbone 2009), and *Picea abies* (Vohník et al. 2007), but also in *Castanea sativa* (Reis et al. 2016). It remains to be shown whether *Meliniomyces* sp. can form true ectomycorrhizae (Hambleton 2005). In the present study, we detected only one species, *Sphaerosporella brunnea*, of the Pezizales order, present on 0.1% of the total sampled ECM root tips. This fungus is a pioneer and opportunist ECM species, often present in nurseries (Sánchez et al. 2014).

Fungal species composition tends to change as stands mature, and older trees tend to support a greater number of fungal symbionts than younger trees (Nara et al. 2003; Twieg et al. 2007). In Portugal, 46 ECM species were reported on root tips of mature *C. sativa* stands (Baptista et al. 2010) and 39 ECM genera in 100-year-old chestnut orchard (Reis et al. 2016). In Italy, 38 (Peintner et al. 2007) and 52 ECM fungal species (Blom et al. 2009) were identified from *C. sativa* root tips. In addition, the investigation developed in central Spain on ECM fungal diversity of *C. sativa*, based on both sporocarp collection and root tips, found 115 ECM fungal species sorted among 30 genera (Álvarez-Lafuente 2015). In the present study, we found 19 genera and 27 ECM fungal species associated with the young chestnut hybrids, which confirm that young plants have a reduced community of fungal symbionts, although the rarefaction curves suggest that we underestimated species diversity.

The concept “succession,” generally applied to patterns of community development that exhibited replacement of species over time (Chapin III et al. 2002), has been used to describe the below-ground changes of ECM fungal community (Mason et al. 1983; Visser 1995). Fungal taxa may be categorized as “early-stage,” “multi-stage” (occurring in all stages), and “late-stage” depending on the host plant age (Twieg et al. 2007). Here, the ECM fungal community associated with the young chestnuts saplings was dominated by *Scleroderma* spp., *C. geophilum*, and *Laccaria* spp*.* for which the highest importance values were found (Fig. 3a–d). *Scleroderma* species are commonly used for inoculation practice in greenhouse nurseries to improve plant performance (Ortega et al. 2004), as well as *Laccaria* spp. (Sinclair et al. 1982), and both are generally considered early-stage fungi, because of their easy dispersal and fast colonization of young root systems of ECM host trees. *Scleroderma* spp. can improve afforestation efforts (Itoo and Reshi 2014) because they are able to tolerate high temperatures and to persist in conditions of drought, thanks to the production of abundant mycelium and rhizomorphs which facilitates water transport (Jeffries 1999). In particular, *Scleroderma* spp*.*, *Hebeloma arenosum*, and *H. sacchariolens* were found in association with the chestnut hybrid clone 111 in both grafted and ungrafted plants, whereas *Inocybe* spp. were found in all the groups. This fact is not surprising; species of *Hebeloma*, *Inocybe*, and *Laccaria* also colonize roots of young *Betula* trees (Mason et al. 1984), and *Inocybe* and *Laccaria* were the first colonizers of *Salix renii* in pioneer conditions (Nara et al. 2003). Interestingly, all *Laccaria* spp. identified in the present study have not been previously recorded in association with chestnuts and have been identified now for the first time in Galicia.

On the other hand, *Lactarius* and *Tricholoma* species, generally dominant in *C. sativa* mature stands (Diamandis and Perlerou 2001; Laganà et al. 2002; Blom et al 2009; Baptista et al. 2010; Polemis et al. 2011; Álvarez-La Fuente 2015; Ambrosio and Zotti 2015; Baptista et al. 2015; Reis et al. 2016), were not found, which suggest that such species can be considered as late-stage fungal symbionts of woody trees. Other late-stage fungi, such as *Russula* and *Boletus*, were rarely found in symbiosis with the experimental saplings. Our data support the hypothesis that saplings are mostly associated with early-stage, generalistic fungal taxa.

To conclude, our study represents a first glance to the fungal partners hosted by young chestnut *Castanea* × *coudercii* hybrids by using molecular methods. Besides the characterization of the ECM fungal community, this investigation also provides a view of the soil saprotrophic fungi, reveals previously undetected fungal species, and highlights for the first time the impact of selected chestnut hybrid clones and grafting on fungal colonization rate. However, further genetic characterization of fungal species diversity and succession is needed, at a more large temporal and spatial scale, to unveil the major processes involved in the distribution of the fungal symbionts in chestnut orchards. Hopefully, the information on the ECM fungal community structure associated with grafted chestnuts hybrids could help in the sustainable management of the traditional chestnut agro-ecosystems, by improving beneficial activities of microorganisms in the soil and rhizosphere (Altieri 1999).

## Supplementary Information

Below is the link to the electronic supplementary material.

Supplementary file1 (DOCX 19 KB)

Supplementary file2 (DOCX 45 KB)

Supplementary file3 (DOCX 16 KB)
