# Supplementary material for: Ectomycorrhizal fungal community structure in a young orchard of grafted and ungrafted hybrid chestnut saplings
Source: Mycorrhiza. 2021 Jan 27;31(2):189–201. doi: 10.1007/s00572-020-01015-0 (PMC7910378; doi:10.1007/s00572-020-01015-0)
Supplement: Supplementary file 1 — Supplementary file1 (DOCX 19 KB) [file 572_2020_1015_MOESM1_ESM.docx]

**Ectomycorrhizal fungal community structure in a young orchard of grafted and ungrafted chestnut hybrid (*Castanea* x *coudercii*)**

Serena Santolamazza-Carbone, Laura Iglesias-Bernabé, Esteban Sinde-Stompel, Pedro Pablo Gallego

| **Soil parameters** | |
| --- | --- |
| **pH** | 4.92±0.10 |
| **Ca^2+^  (cmol/Kg)** | 1.20±1.05 |
| **Mg^2+^  cmol/Kg)** | 0.46±0.26 |
| **Na^+^  (cmol/Kg)** | 0.13±0.03 |
| **K^+^  (cmol/Kg)** | 0.20±0.03 |
| **Al ^3+^  (cmol/Kg)** | 1.48±0.49 |
| **CEC** | 3.46±0.83 |
| **C (gKg ^-1^)** | 32.33 ± 4.12 |
| **N (gKg ^-1^)** | 2.40 ± 0.31 |
| **C/N** | 13 ± 0.7 |
| **Ca^2+^  / Mg^2+^** | 3.27±2.96 |
| **Ca^2+^  / K^+^** | 6.35±6.05 |
| **K^+^  / Mg^2+^** | 0.54±0.30 |
| **Organic matter** | 55.23 ± 7.11 |

**Electronic Supplementary material S1**

Soil parameters (mean ± SD). Twenty-four soil samples were collected in the experimental orchard in Galicia (NW Spain). CEC = Cation Exchange Capacity.
