# Supplementary material for: Ectomycorrhizal fungal community structure in a young orchard of grafted and ungrafted hybrid chestnut saplings
Source: Mycorrhiza. 2021 Jan 27;31(2):189–201. doi: 10.1007/s00572-020-01015-0 (PMC7910378; doi:10.1007/s00572-020-01015-0)
Supplement: Supplementary file 2 — Supplementary file2 (DOCX 45 KB) [file 572_2020_1015_MOESM2_ESM.docx]

**Ectomycorrhizal fungal community structure in a young orchard of grafted and ungrafted chestnut hybrid (*Castanea* x *coudercii*)**

Serena Santolamazza-Carbone, Laura Iglesias-Bernabé, Esteban Sinde-Stompel, Pedro Pablo Gallego


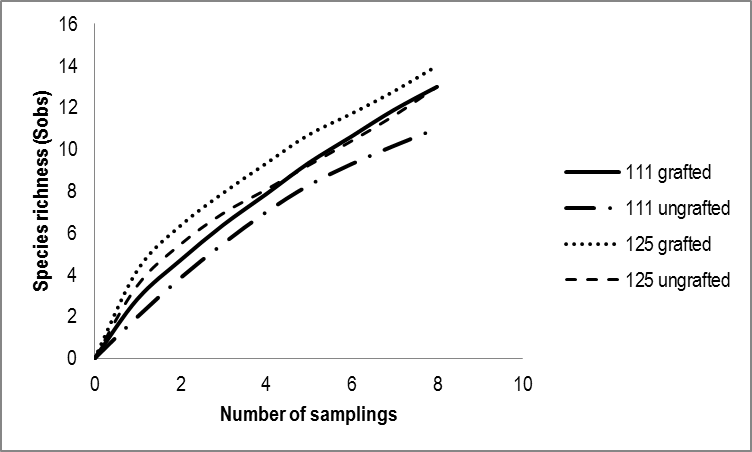


**A**

|  | 111 grafted | 111 ungrafted | 125 grafted | 125 ungrafted |
| --- | --- | --- | --- | --- |
| Sobs | 13 | 11 | 14 | 13 |
| Sest | 32.69 | 13.90 | 30.20 | 52.37 |
| Sobs/Sest | 0.40 | 0.80 | 0.46 | 0.25 |

**B**

**Electronic Supplementary material S2**

Rarefied species accumulation curves of the grafted and ungrafted clones 111 and 125 of the chestnut hybrid *Castanea* x *coudercii* (A). Each curve represents the mean of 100 randomizations of sampling order. The table (B) shows the observed (Sobs) and estimated (Sest) ECM fungal species richness and the ratio between them by using the Chao2 method.
