# Supplementary material for: Ectomycorrhizal fungal community structure in a young orchard of grafted and ungrafted hybrid chestnut saplings
Source: Mycorrhiza. 2021 Jan 27;31(2):189–201. doi: 10.1007/s00572-020-01015-0 (PMC7910378; doi:10.1007/s00572-020-01015-0)
Supplement: Supplementary file 3 — Supplementary file3 (DOCX 16 KB) [file 572_2020_1015_MOESM3_ESM.docx]

**Ectomycorrhizal fungal community structure in a young orchard of grafted and ungrafted chestnut hybrid (*Castanea* x *coudercii*)**

Serena Santolamazza-Carbone, Laura Iglesias-Bernabé, Esteban Sinde-Stompel, Pedro Pablo Gallego

| Order | Family | Identification (Blast search) | Similarity (%) | Size (bp) | GenBank accession | Trophism |
| --- | --- | --- | --- | --- | --- | --- |
| Agaricales | Crepidotaceae | *Crepidotus* sp. (MF461345) | 100 | 809 | MN651959 | saprotrophic |
|  | Entolomataceae | *Clitopilus scyphoides* (KC176282) | 99 | 819 | MN945081 | saprotrophic |
|  | Strophariaceae | *Hypholoma fasciculare* (FJ430716) | 99 | 812 | MN945142 | saprotrophic |
| Cantharellales | Ceratobasidiaceae | *Ceratobasidium* sp. *(*KP281807) | 99 | 768 | MN945145 | wood decay fungus |
| Helotiales | Hyaloscyphaceae | *Meliniomyces* sp. (FN66923) | 99 | 569 | MN945082 | ericoid mycorrhiza |
| Hymenochaetales | Schizoporaceae | *Schizopora* sp. (MF161274) | 99 | 689 | MN947225 | wood decay fungus |
| Polyporales | Ganodermataceae | *Ganoderma adspersum* (EF660009) | 99 | 763 | MN945139 | wood decay fungus |
|  | Gelatoporiaceae | *Obba rivulosa* (JQ027728) | 99 | 481 | MN944925 | saprotrophic |
|  | Meruliaceae | *Phlebia acerina* (LN611083) | 99 | 738 | MN945144 | saprotrophic |
|  | Meruliaceae | *Phlebia livida* (HQ153424) | 98 | 718 | MN945152 | saprotrophic |
|  | Meruliaceae | *Scopuloides hydnoides* (LN611118) | 98 | 729 | MN944926 | saprotrophic |
|  | Phanerochaetaceae | *Phlebiopsis gigantea* (MF476018) | 99 | 746 | MN945141 | saprotrophic |
| Russulales | Peniophoraceae | *Peniophora rufomarginata* (MH857639) | 99 | 729 | MN947226 | saprotrophic |

**Electronic Supplementary Material S3**

Non-ECM fungi found in association with chestnut hybrid clones. Taxonomical identity was ascertained by direct sequencing of the ITS regions and BLAST search.
